# Use of SNOMED CT in Large Language Models: Scoping Review

**DOI:** 10.2196/62924

**Published:** 2024-10-07

**Authors:** Eunsuk Chang, Sumi Sung

**Affiliations:** 1 Republic of Korea Air Force Aerospace Medical Center Cheongju Republic of Korea; 2 Department of Nursing Science Research Institute of Nursing Science Chungbuk National University Cheongju Republic of Korea

**Keywords:** SNOMED CT, ontology, knowledge graph, large language models, natural language processing, language models

## Abstract

**Background:**

Large language models (LLMs) have substantially advanced natural language processing (NLP) capabilities but often struggle with knowledge-driven tasks in specialized domains such as biomedicine. Integrating biomedical knowledge sources such as SNOMED CT into LLMs may enhance their performance on biomedical tasks. However, the methodologies and effectiveness of incorporating SNOMED CT into LLMs have not been systematically reviewed.

**Objective:**

This scoping review aims to examine how SNOMED CT is integrated into LLMs, focusing on (1) the types and components of LLMs being integrated with SNOMED CT, (2) which contents of SNOMED CT are being integrated, and (3) whether this integration improves LLM performance on NLP tasks.

**Methods:**

Following the PRISMA-ScR (Preferred Reporting Items for Systematic Reviews and Meta-Analyses extension for Scoping Reviews) guidelines, we searched ACM Digital Library, ACL Anthology, IEEE Xplore, PubMed, and Embase for relevant studies published from 2018 to 2023. Studies were included if they incorporated SNOMED CT into LLM pipelines for natural language understanding or generation tasks. Data on LLM types, SNOMED CT integration methods, end tasks, and performance metrics were extracted and synthesized.

**Results:**

The review included 37 studies. Bidirectional Encoder Representations from Transformers and its biomedical variants were the most commonly used LLMs. Three main approaches for integrating SNOMED CT were identified: (1) incorporating SNOMED CT into LLM inputs (28/37, 76%), primarily using concept descriptions to expand training corpora; (2) integrating SNOMED CT into additional fusion modules (5/37, 14%); and (3) using SNOMED CT as an external knowledge retriever during inference (5/37, 14%). The most frequent end task was medical concept normalization (15/37, 41%), followed by entity extraction or typing and classification. While most studies (17/19, 89%) reported performance improvements after SNOMED CT integration, only a small fraction (19/37, 51%) provided direct comparisons. The reported gains varied widely across different metrics and tasks, ranging from 0.87% to 131.66%. However, some studies showed either no improvement or a decline in certain performance metrics.

**Conclusions:**

This review demonstrates diverse approaches for integrating SNOMED CT into LLMs, with a focus on using concept descriptions to enhance biomedical language understanding and generation. While the results suggest potential benefits of SNOMED CT integration, the lack of standardized evaluation methods and comprehensive performance reporting hinders definitive conclusions about its effectiveness. Future research should prioritize consistent reporting of performance comparisons and explore more sophisticated methods for incorporating SNOMED CT’s relational structure into LLMs. In addition, the biomedical NLP community should develop standardized evaluation frameworks to better assess the impact of ontology integration on LLM performance.

## Introduction

### Background

The recent emergence of large language models (LLMs), exemplified by Bidirectional Encoder Representations from Transformers (BERT) [1] and GPT [2], has significantly advanced the capabilities of machines in natural language understanding (NLU) and natural language generation (NLG). Despite achieving state-of-the-art performance on a range of natural language processing (NLP) tasks, LLMs exhibit a deficiency in knowledge when confronted with knowledge-driven tasks [3]. These models acquire factual information from extensive text corpora during training, embedding this knowledge implicitly within their numerous parameters and consequently posing challenges in terms of verification and manipulation [4]. Moreover, numerous studies have demonstrated that LLMs struggle to recall facts and frequently encounter hallucinations, generating factually inaccurate statements [5,6]. This poses a significant obstacle to the effective application of LLMs in critical scenarios, such as medical diagnosis and legal judgment [7].

Efforts have been made to address the black box nature of LLMs and mitigate potential hallucination problems. Approaches include enhancing language model (LM) veracity through strategies such as retrieval chain-of-thought prompting [8] and retrieval-augmented generation [9]. Another significant avenue involves integrating knowledge graphs (KGs) or ontologies into LMs using triple relations or KG subgraphs [7,10]. KGs, renowned for their excellence in representing knowledge within a domain, can provide answers when combined with LMs [11], making them valuable for common sense–based reasoning and fact-checking models [12]. However, LLMs often face challenges when trained and tested predominantly on general-domain datasets or KGs, such as Wikipedia and WordNet [13], making it difficult to gauge their performance on datasets containing biomedical texts. The differing word distributions in general and biomedical corpora pose challenges for biomedical text mining models [14].

Biomedicine-specific KGs may be a potential solution to the abovementioned problems. In the biomedical domain, KGs, also known as ontologies, are relatively abundant, with the Unified Medical Language System (UMLS) [15] being one of the most frequently used ontologies [16]. The UMLS serves as a thesaurus for biomedical terminology systems such as the Medical Subject Headings, International Classification of Diseases, Gene Ontology, Human Phenotype Ontology, and SNOMED CT, all curated and managed by the United States National Library of Medicine.

Among UMLS member terminologies, SNOMED CT stands out as the most comprehensive biomedical ontology, encompassing a wide range of biomedical and clinical entities, including signs, symptoms, diseases, procedures, and social contexts [17]. These entities are represented by concepts (clinical ideas), descriptions (human-readable terms linked to concepts), and relations (comprising hierarchical *is-a* relations and horizontal attribute relations). As SNOMED CT is increasingly integrated into electronic health record (EHR) systems, as required by the Fast Healthcare Interoperability Resource (FHIR) to ensure interoperability among health care institutions [18], terminology servers supporting SNOMED CT have become ubiquitous. With its ready availability across health care institutions, SNOMED CT has gained attention as a knowledge source or ontology for representing biomedical and clinical knowledge [17]. In this case, the abstract model of SNOMED CT is used to describe and store biomedical facts in a hierarchical and structured manner, readily available across health care institutions.

Integrating SNOMED CT into LLMs holds significant potential for advancing various aspects of health care and biomedical research. By incorporating the comprehensive and structured biomedical knowledge from SNOMED CT, LLMs can better understand medical terminology, relationships between clinical concepts, and domain-specific context, potentially reducing errors and hallucinations when understanding or generating biomedical texts. This integration could enhance clinical decision support systems, improve the accuracy of automated coding and billing processes, facilitate more precise information retrieval from medical literature, and support the development of personalized medicine approaches. Furthermore, it may enable more accurate NLP of clinical notes and medical records, potentially leading to improved patient care and outcomes through better data analysis and insights.

### Objectives

This scoping review aimed to examine the use of SNOMED CT as a knowledge source to be incorporated into LLMs, specifically focusing on the methodology of integrating these 2 modalities. This review sought to answer the following research questions: (1) What are the dominant types and components of LLMs being integrated with SNOMED CT? (2) Which contents of SNOMED CT (ie, descriptions, relations, or entity classes) are being integrated into LLMs? and (3) Does the integration of SNOMED CT into LLMs improve the performance on NLP tasks in terms of NLU and NLG? Answers to these questions could suggest future methodological approaches for more effectively integrating human-engineered knowledge into LLMs.

## Methods

This scoping review was guided by the PRISMA-ScR (Preferred Reporting Items for Systematic Reviews and Meta-Analyses extension for Scoping Reviews) framework, which outlines the recommended steps and reporting standards for conducting scoping reviews (Multimedia Appendix 1) [19].

### Study Identification

We defined LLMs as transformer-based LMs pretrained on large-scale corpora [20] (Multimedia Appendix 2). Given that transformer-based models currently dominate in the field and are likely to continue doing so in the coming years, reviewing other LMs, such as recurrent neural networks and more conventional statistical models, does not hold scientific significance for current and future applications. Therefore, focusing on transformer-based models allows a more cohesive and in-depth analysis of the most relevant and cutting-edge techniques in the field.

To explore scientific literature describing transformer-based models, we conducted our literature search on ACM Digital Library, ACL Anthology, IEEE Xplore, PubMed, and Embase on March 12, 2024, using the following query terms: (1) (“language *model” OR “pretrained *model” OR “language processing” OR “embedding”) AND (“SNOMED” OR “Unified Medical Language System” OR “UMLS” OR “*medical”) AND (“knowledge graph” OR “ontolog*” OR “knowledge*base” OR “knowledge infusion”) and (2) (“SNOMED”) AND (“large language model” OR “BERT” OR “GPT”). Queries were modified according to the bibliographic databases when necessary. Queries were designed to search for articles published from 2018 to 2023. The start date of the query was set to 2018 when BERT, the first transformer-based LM to gain widespread adoption, was introduced, marking the beginning of significant research into transformer-based LLMs.

### Study Selection

Articles were extracted from ACM Digital Library, ACL Anthology, IEEE Xplore, PubMed, and Embase. Duplicates were removed, and 2 authors (SS and EC) examined the full text of the retrieved articles for the presence of the term “SNOMED.” We prioritized a full-text search first before title and abstract review because many potentially eligible papers do not explicitly mention “SNOMED” in their titles or abstracts. To be eligible for our review, articles had to have SNOMED CT incorporated into NLP pipelines, which encompass processes from text cleansing through pretraining and inference to model evaluation, specifically for tasks involving NLU and NLG. We then further excluded studies that met ≥1 of the following criteria: (1) published in languages other than English; (2) categorized as reviews, surveys, keynotes, or editorial articles; (3) did not incorporate SNOMED CT at any stage of the NLP pipeline; (4) aimed to create, develop, enrich, or enhance ontologies or graphs; (5) did not involve the processing of natural language (NL) text; or (6) solely used SNOMED CT codes for retrieving patients of interest from EHRs or for annotating instances with SNOMED CT codes as gold-standard target labels for LM training.

### Result Synthesis

Through discussions and qualitative assessments, we analyzed the included articles according to the following characteristics: chronological and geographic publication trends, baseline LLM and its output, dataset used for training and testing the model, methods for integrating SNOMED CT into the LLM, and the model’s end task and performance (Textbox 1).

**Methods for synthesizing the review.**

**Synthesis of results**

- Chronological and geographic publication trends
- Baseline large language model (LLM) and its output
- Dataset used for training and testing the model
- Methods for integrating SNOMED CT into the LLM (methodologies for knowledge graph [KG]–enhanced LLMs [7])
  - KG-enhanced LLM pretraining: works that apply KGs during the pretraining stage and improve the knowledge expression of LLMs
  - KG-enhanced LLM interpretability: works that use KGs to understand the knowledge learned by LLMs and interpret the reasoning process of LLMs
  - KG-enhanced LLM inference: research that uses KGs during the inference stage of LLMs, which enables LLMs to access the latest knowledge without retraining
- End task and performance
End task natural language understanding: entity recognition or typing, entity or relation extraction, document classification, question answering (multiple choice), and inference End task natural language generation: text summarization, question answering (short or essay answers), translation, and dialogue generation Performance analysis: nominal percentage gains in performance after SNOMED CT integration

We elucidated the methodology for incorporating SNOMED CT into NLP pipelines following the categorization methods previously outlined by Pan et al [7]. These methods categorized methodologies for KG-enhanced LLMs into three distinctive types: (1) KG-enhanced LLM pretraining, (2) KG-enhanced LLM interpretability, and (3) KG-enhanced LLM inference. The end tasks of LLMs after SNOMED CT integration included NLU and NLG. Regarding the performance analysis, we presented the nominal percentage gains in performance after SNOMED CT integration without analyzing their statistical significance, as most studies did not perform statistical significance testing. We refrained from conducting direct study-to-study comparisons due to concerns about the heterogeneity of testing corpora and evaluation metrics across different studies.

## Results

### Selected Papers

**Figure 1:**
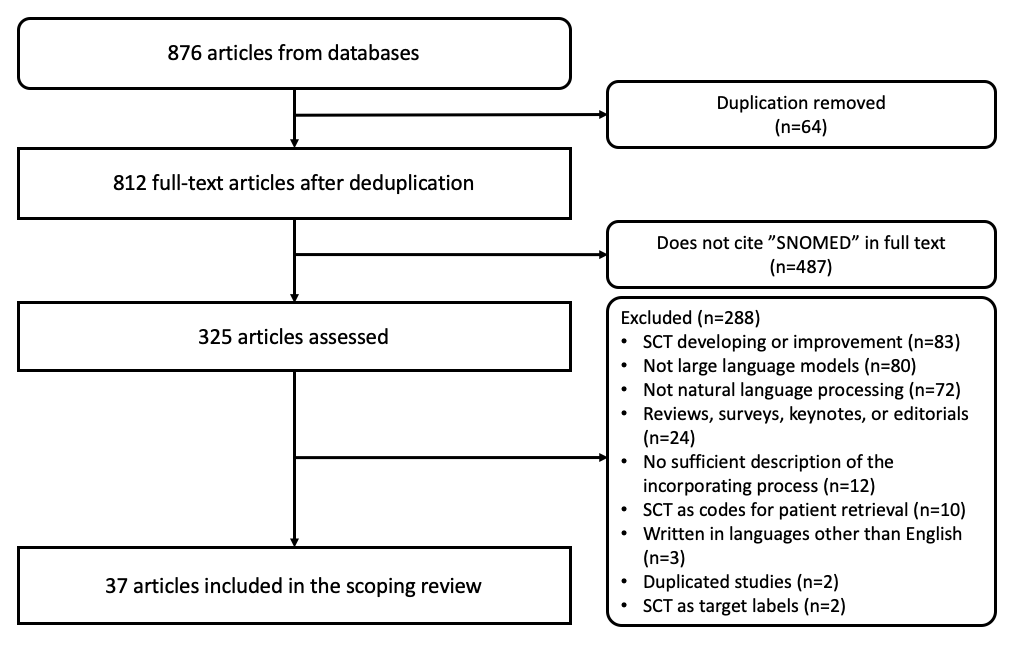
PRISMA (Preferred Reporting Items for Systematic Reviews and Meta-Analyses) flow diagram of article selection. SCT: SNOMED CT.

The query yielded 876 articles from the 5 bibliographic databases, with 634 (72.4%) obtained from the first query and 242 (27.6%) from the second query (Figure 1). After the removal of duplicates, 812 (92.7%) articles were reviewed to check whether the term “SNOMED” was mentioned in their full texts. A total of 325 (37.1%) articles were then reviewed according to the inclusion and exclusion criteria. Consequently, 37 (4.2%) publications were finally selected for the scoping review (Figure 1). The characteristics of the individual papers and other features, including the language of used datasets and SNOMED CT descriptions, other ontologies used, and the types of entities represented by SNOMED CT, are detailed in Multimedia Appendix 3.

### Chronological and Geographic Publication Trends

Table 1 presents the publication trends noted in the review. Although our literature search covered publications from 2018 onward, no studies published in 2018 were included in the final review. The largest volume of studies was published in 2022 (13/37, 35%), followed by those published in 2020 (10/37, 27%).

When the number of countries was counted according to the first authors’ institutional affiliations, the largest number of studies was noted to originate from the United States (10/37, 27%). While most of the studies (26/37, 70%) were conducted in countries that are members of SNOMED International, some were performed in nonmember countries such as Bulgaria and China, where separate license fees and in-house translation of SNOMED CT descriptions to the local language were required.

**Table 1:** Chronological and geographic publication trends among the included studies.

Study characteristics		Studies
**Publication year**		
	2019	[21-23]
	2020	[24-33]
	2021	[34-36]
	2022	[37-49]
	2023	[50-57]
**Countries**		
	Australia	[26,35]
	Bulgaria	[34,52]
	Canada	[55]
	China (including Hong Kong)	[28,38,39,41,43,45,48,50,56]
	Germany	[47,51]
	India	[22,31,32]
	Israel	[53]
	Spain	[21,29,30,37,40]
	United Kingdom	[54,57]
	United States	[23-25,27,33,36,42,44,46,49]
**Publication type**		
	Journal paper	[23-26,36,42-46,50,55-57]
	Conference paper	[21,22,27-35,37-41,45,47-49,51-54]

### Baseline LLMs and Their Outputs

Most of the included studies (27/37, 73%) used BERT and its variants as the baseline LLMs for NLU and NLG tasks. Variants such as RoBERTa [58] and ALBERT [59] were also used to address BERT’s relatively small training corpora and long training time [31,37,38,50,53]. To overcome the limited applicability of these general-purpose LLMs to biomedical texts, many studies (13/37, 35%) used LLMs trained on large-scale biomedical corpora, such as BioBERT [14] and PubMedBERT [60], which were trained on PubMed articles, and ClinicalBERT [61] and EHRBERT [23], which were trained on clinical notes. SapBERT [62], initialized by PubMedBERT, was further fine-tuned using contrastive learning with UMLS synonyms to better accommodate SNOMED CT synonym descriptions [44,47]. To support biomedical NLP tasks in languages other than English, LLMs trained on corpora in those languages were also adopted, such as medBERT.de [63], designed specifically for the German medical domain [51], and ERNIE-health, pretrained from Chinese medical records [41]. Aside from these BERT-based models, GPT emerged as a new baseline LLM since 2023. Makhervaks et al [53] used BioGPT [64], whose decoder was pretrained on biomedical corpora, to enhance the generation of artificial sentences. In addition, Xu et al [55] used GPT-3.5 for ranking suggested annotation terms in their study (Table 2).

A primary assertive role of LLMs was representing biomedical entities from text data. While most proposed methods produced embedding vectors to convey contextual information about the biomedical entities that appeared in texts, Kalyan and Sangeetha [31] introduced a Siamese RoBERTa model to generate concept vectors from synonym relationships defined by SNOMED CT. These basic outputs of LLMs might undergo additional task-specific layers to perform desired end tasks, which will be discussed later. Beyond producing embedding representations of entities, some studies required LLMs to perform classification or ranking tasks after fine-tuning, predicting the most likely relevant standard concepts [23,24,26,34,41,55], entity types [35,38,51], sentences [49,53], or matched foreign language words, enabling machine translation [28-30,39]. LLMs with encoder-decoder architectures, such as BART [65], were used for dedicated NLG tasks [32,57].

**Table 2:** Large language models used in the included studies.

Base and fine-tuned models			Studies
**BERT^a^**			
	Vanilla BERT	[22,24,26,27,33,40,42-44,50,53,54,56,57]	
	RoBERTa	[31,37,38,50]	
	ALBERT	[53]	
	ELECTRA	[53]	
	DeBERTa	[53]	
	mBERT	[37,45]	
	BioBERT	[27,33,34,46,48,49,52]	
	ClinicalBERT	[25,33,35,36]	
	PubMedBERT	[45,46]	
	SAPBERT	[44,47]	
	EHRBERT	[23]	
	SciBERT	[46]	
	BioELECTRA	[53]	
	German BERT models	[51]	
**GPT**			
	GPT-3.5	[55]	
	BioGPT	[53]	
	BART	[57]	
**Transformer neural networks**			
	Transformer NMT^b^ model	[21,28-30,39]	
	Denoising autoencoder	[32]	
**ERNIE^c^**			
	ERNIE-health	[41]	

^a^BERT: Bidirectional Encoder Representations from Transformers.

^b^NMT: neural machine translation.

^c^ERNIE: Enhanced Language Representation with Informative Entities.

### Data for Training and Testing Models

When using general-domain LLMs, authors deployed additional fine-tuning or pretraining on biomedical corpora to better adapt their models for biomedical NLP tasks. The pretraining corpora included PubMed or MEDLINE articles [28,30,38,39,46] and other publicly available datasets, such as Wikipedia articles [29] and tweets [37] related to biomedical topics. Synthetic sentences were also used to address data scarcity, which was generated based on SNOMED CT descriptions or relations [21,29].

While some studies (8/37, 22%) used real-world clinical narrative records [21,30,48,52] or customized (ie, manually annotated by researchers) data [25,27,41,56] for testing their models, most of the studies (29/37, 78%) used publicly available datasets, especially when researchers were participating in shared task competitions or dealing with English texts. CADEC [66] and PsySTAR [67], open datasets built from drug review posts in which concept mentions were mapped to SNOMED CT concepts, were used for validating and testing concept normalization models [31,45]. The Medical Concept Normalization (MCN) corpus, drawn from discharge summaries annotated using SNOMED CT and RxNorm concepts, was experimented on by concept normalization models [24,26]. The WMT corpora, provided by the annual Conference on Machine Translation Shared Tasks, were used to test multilingual machine translation tasks by participating researchers [28,29,39]. Makhervaks et al [53] and Chopra et al [22] used sentence pairs in the MedNLI corpus [68], annotated by medical doctors into 3 categories—contradictory, entailing, and neutral—for NL inference tasks. The MedMentions corpus [69] identifies >350,000 mentions from >4000 PubMed abstracts, linking them to the UMLS concepts; it was used in the studies by Zotova et al [40] and Dong et al [54], in which SNOMED CT was loaded onto the UMLS. The ShARe/CLEF 2013 corpus [70] consists of deidentified clinical notes annotated with disease mentions using the SNOMED CT subset of the UMLS; it was used for testing concept normalization tasks [44,54].

### SNOMED CT Content Integration Into NLP Pipelines

#### Overview

While the categorization methods by Pan et al [7] pertained to the integration of LLMs with general-purpose KGs, we treated SNOMED CT as a specified form of KG. Their third category—KG-enhanced LLM interpretability—was omitted due to the lack of relevant studies in our review. In addition, we found no studies that fit into the subcategories “Integrating KGs into Training Objectives” (under “KG-enhanced LLM pretraining”) and “Dynamic Knowledge Fusion” (under “SNOMED CT–enhanced LLM inference”). The overarching categorization of all included methods is shown in Textbox 2.

**Summarized categorizations of SNOMED CT–incorporated large language model (LLM) methods (allowed duplicated counting of studies).**

**Category and subcategory**

- SNOMED CT–enhanced LLM pretraining
  Integrating SNOMED CT into LLM inputs (n=28, 76%)
  Integrating SNOMED CT into additional fusion modules (n=5, 14%)
- SNOMED CT–enhanced LLM inference
  Retrieval-augmented knowledge fusion (n=5, 14%)

#### Integration of SNOMED CT Into LLM Inputs

##### Overview

Research in this area concentrated on developing new training objectives for LLMs that incorporate knowledge awareness. More specifically, this line of research aimed to incorporate relevant portions or subsets of SNOMED CT as additional input to LLMs during training. Because a disproportionately large number of included studies (28/37, 76%) fell into this category, we analyzed the methodology by two additional themes: (1) the content of SNOMED CT that was integrated into an LLM and (2) the part of the NLP pipeline into which the aforementioned content was incorporated. After qualitative analysis of the included articles and heuristic discussions among reviewers, we categorized the former theme into descriptions (including descriptions of synonyms), relations, and entity types (classes) and the latter theme into encoders and training data. SNOMED CT contents could be incorporated into LLM encoders either as embedding vectors or as annotations or tags when incorporated into the training corpus.

Table 3 shows the distribution of models across SNOMED CT contents and NLP pipelines, allowing for duplicated counting of a single study if it adopted ≥2 methods.

**Table 3:** Distributions of models across SNOMED CT contents and natural language processing (NLP) pipelines.

SNOMED CT content integrated into the NLP pipeline	Part of the NLP pipeline where SNOMED CT contents were integrated into	
	Encoder (as vector embedding)	Training corpora (as annotated text)
Description	[31,35,41,43,44,54]	[21,23,24,28-30,32,34,39,40,47-50,52,54,57]
Relation	[31,45]	[21,34,40,52,53]
Entity type (class)	—^a^	[25,38,42,51]

^a^Not available.

##### Integration of SNOMED CT Descriptions

Vector representations of SNOMED CT concept descriptions were created to facilitate seamless fusion into LLM encoders. The vectors for SNOMED CT description embeddings were used to calculate cosine similarity between the original mentions and SNOMED CT descriptions for concept normalization tasks [35,41,43,54].

**Figure 2:**
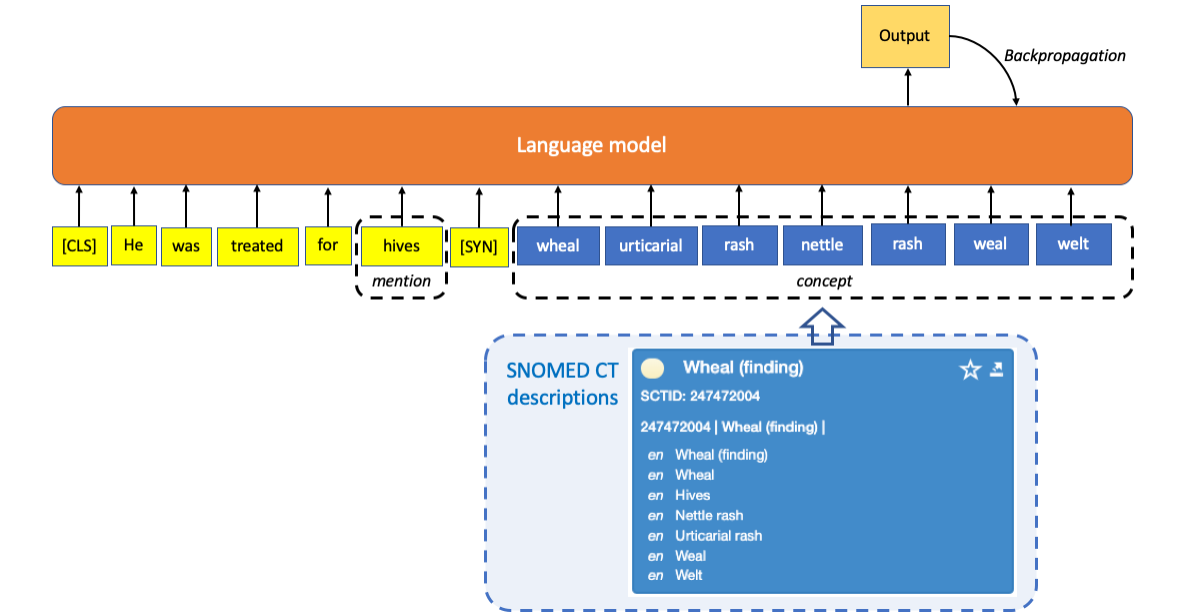
Integrating SNOMED CT descriptions into large language models. CLS: classification; SYN: synonym.

Instead of transforming text descriptions into vector embeddings, NL description texts were directly added to training corpora to expand the size of in-domain vocabulary (Figure 2). The description texts of synonyms were either concatenated in the training corpora before being input into an LLM for pretraining [24,47,49,54,57] or they replaced the original entity mentions in the text with standardized terms [32,48]. The descriptions of SNOMED CT codes were also prepended to the word sequences as classifier tokens for LLM pretraining [23]. The multilingual feature of SNOMED CT descriptions was exploited to address the limited availability of training datasets in foreign languages by adding the translated SNOMED CT descriptions into the training corpora [28-30,39,50].

##### Integration of SNOMED CT Relations

**Figure 3:**
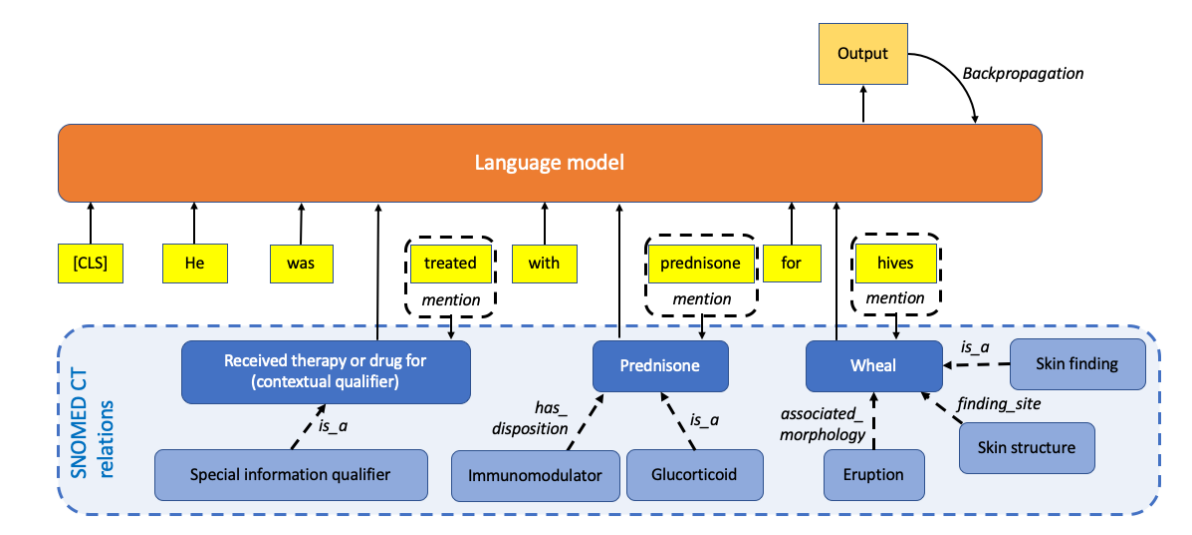
Integrating SNOMED CT relations into large language models. CLS: classification.

This line of research introduced relevant subgraph information of SNOMED CT, representing SNOMED CT relations as graph edges, into LLMs (Figure 3). Kalyan and Sangeetha [31] encoded SNOMED CT concept descriptions to generate concept embedding vectors and learn representation vectors of concept mentions in the text, further improving the representations by retrofitting the target concept vectors with SNOMED CT synonym relations. CODER [45] used KG embedding methods such as DistMult and ANALOGY [71] to learn relational knowledge from SNOMED CT, enabling the quantification of term-relation-term similarity as well as term-term similarity.

A different approach was taken to introduce textual relation triplets defined by SNOMED CT to expand the size of training corpora. Soto et al [21] exploited the relations defined in SNOMED CT, such as *is_a* and *occurs_in*, to generate synthetic training corpora. Relations defined in SNOMED CT were also used to apply weak supervision to sentence pairs extracted from PubMed to establish contradiction labels in the dataset [53]. Other authors exploited the existing mappings to other ontologies (eg, International Classification of Diseases-10 and UMLS) to enrich the training corpus with the description texts from the linked ontology concepts [34,40,52].

##### Integration of SNOMED CT Entity Types

**Figure 4:**
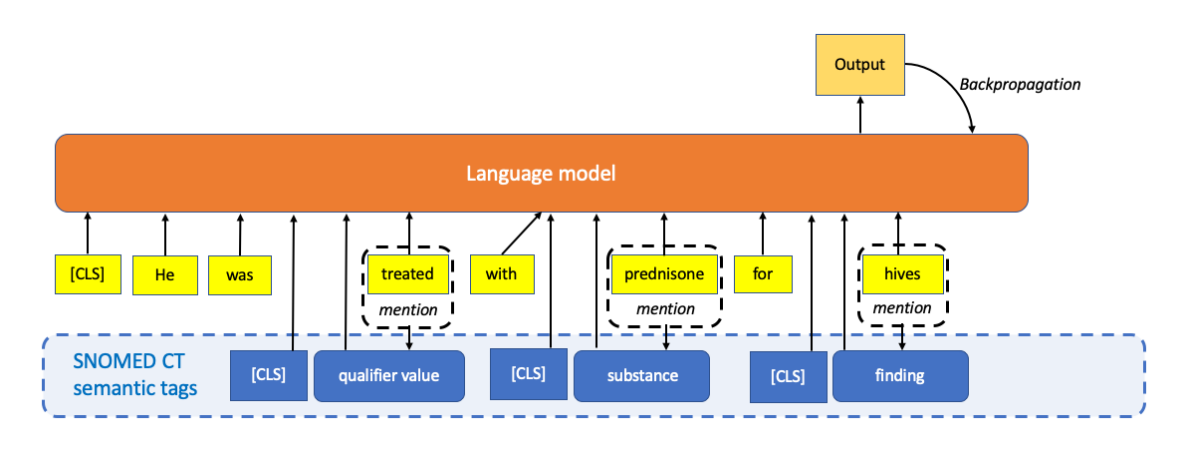
Integrating SNOMED CT entity type information into large language models. CLS: classification.

The type of entities was incorporated into training corpora by distantly labeling the identified entities with SNOMED CT semantic tags (eg, diseases and chemicals; Figure 4) [25,38]. In other studies, training corpora were annotated with SNOMED CT top-level hierarchies [51] or subclasses of top-level hierarchies [42] to label sentences per their respective tasks.

#### Integration of SNOMED CT Into Additional Fusion Modules

**Figure 5:**
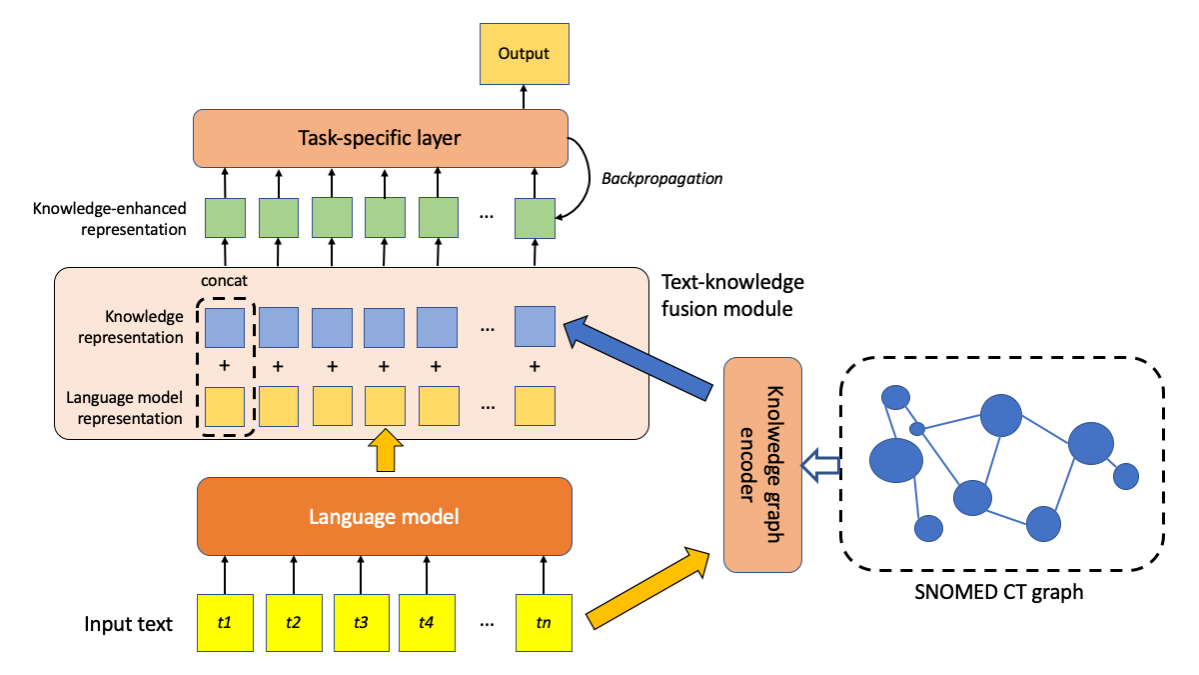
Integrating SNOMED CT into additional fusion modules.

In this approach, concept information was processed separately before being concatenated and fused with the LLM embedding output (Figure 5). Authors created knowledge-directed embeddings using SNOMED CT graphs, where concepts were represented as nodes and relations as edges, and concatenated them with the LLM contextual embeddings. The merged representations of text and graph embeddings were then passed through a task-specific knowledge fusion module to achieve end tasks such as semantic similarity measurement [36,46], classification [22,27], and question answering [33,46]. To represent the graph information of SNOMED CT concepts, Chang et al [36] used a graph convolutional network [72] for encoding node features and edges. Chopra et al [22] proposed the Bio-MTDDN model, which introduced the shortest path information between corresponding SNOMED CT concepts into knowledge-directed embeddings.

#### Retrieval-Augmented Knowledge Fusion

**Figure 6:**
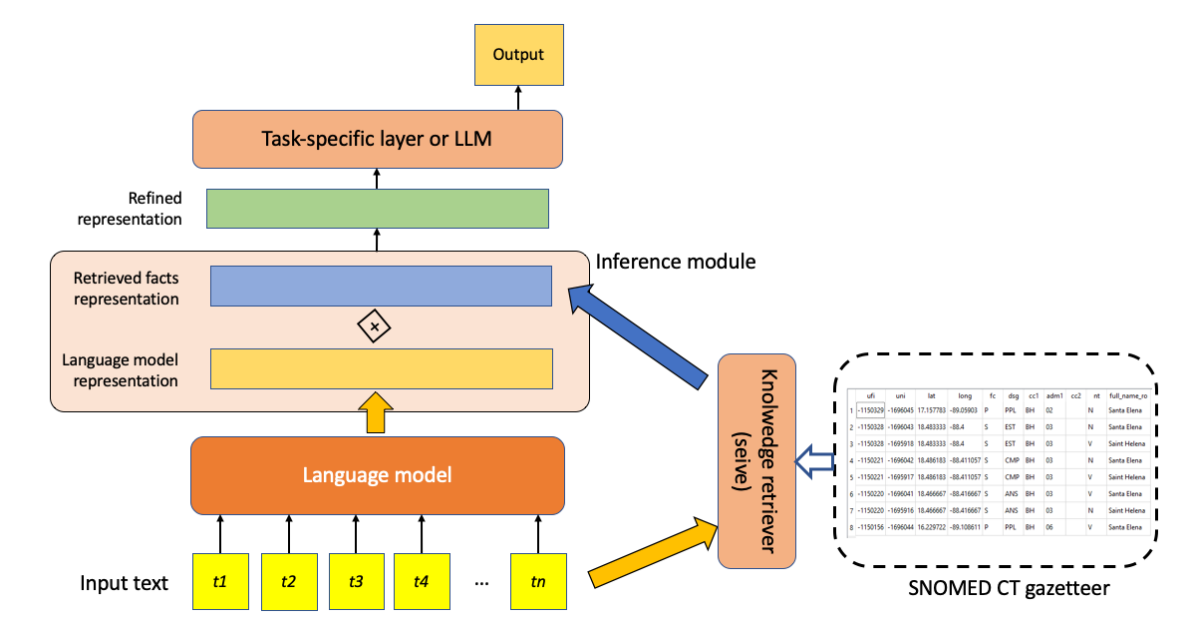
Retrieval-augmented knowledge fusion. LLM: large language model.

In this approach, SNOMED CT was located outside LLMs as a fact-consulting knowledge base, injecting knowledge during inference (Figure 6). The module functioned as a gazetteer (dictionary), matching mentions in texts against the dictionary of SNOMED CT descriptions to filter out irrelevant entities from the models and map textual mentions to the most likely SNOMED CT concepts [24,26,37,55,56]. These methods primarily concentrated on entity recognition and question answering, capturing both textual semantic meanings and up-to-date real-world knowledge.

### End Task and Performance Gain After SNOMED CT Integration

#### Overview

Most of the included studies (30/37, 81%) focused on NLU tasks, such as entity typing and classification. NLG tasks, including translation and summarization, were also attempted by a substantial number of studies (9/37, 24%), often involving various NLU pipelines before producing the final text output. Therefore, notably, works on NLU may also appear in the NLG category. Herein, we also compared the performance of models integrated with SNOMED CT to that of their counterparts without SNOMED CT integration.

#### NLU Tasks

##### Entity Extraction and Typing

Entity typing or named entity recognition tasks aim to detect specific types of entities by identifying the spans of their mentions in the text. These can be regarded as multiclassification tasks, where the number of classes is arbitrarily chosen by researchers. To fine-tune LLMs for type classification, authors annotated entities in texts by matching domain gazetteer strings (eg, “BIO” tagging scheme) [37,38,49] or using off-the-shelf automatic concept extractors [27]. The identified entities were then classified into human-annotated entity types [37,38] or topmost nodes in the SNOMED CT hierarchies [27,51]. In addition to typing individual entities, extraction and typing of relations between 2 entities were also attempted to align the detected entities with FHIR resources [25], such as protein to chemical and gene to disease [46] as well as disease to inflicted family members [35].

Many researchers did not conduct a comparative performance analysis of their SNOMED CT–integrated models against out-of-domain vanilla models. Among the few researchers who reported such comparisons, Jha and Zhang [46] demonstrated a gain in the *F*_1_-score after the integration of SNOMED CT, while Montañés-Salas et al [37] found a positive impact only on recall (Table 4).

**Table 4:** Percentage performance gain in biomedical entity typing tasks after SNOMED CT integration into large language models.

Studies	*F*_1_-score gain (%)	Precision gain (%)	Recall gain (%)	AUC^a^ gain (%)
Montañés-Salas et al [37] (Best 2 model)	−0.11 (0.899→0.898)	−7.97 (0.928→0.854)	+8.60 (0.872→0.947)	—^b^
Jha and Zhang [46] (PubMedBERT on BC2GM)	+4.08 (0.80982→0.84287)	—	—	—

^a^AUC: area under the receiver operating characteristic curve.

^b^Not available.

##### Classification

We defined classification tasks as occurring at the sentence or document level, rather than at the word, entity, or phrase level. When classification tasks were implemented, semantic similarity [36] or the conditional probability of a positive case [22,33,53] was calculated, and the case was categorized as positive if the probability exceeded a threshold. Binary classification was performed to determine whether a sentence pair was entailed [33], contradictory [22,53], or similar [36]. Multilabel classification was conducted to categorize utterances by clinical encounter components, such as symptoms, complaints, and medications [27]; social determinants of health [42]; or narrators’ intent [48].

Table 5 shows the percentage performance gain after SNOMED CT integration in classification tasks. While Yadav et al [33] and Zhang et al [48] estimated the performance of their models based on the *F*_1_-score, precision, and recall, Khosla et al [27] and Makhervaks et al [53] measured performance in terms of the area under the receiver operating characteristic curve, which improved by 0.87% to 14.83% after the integration of SNOMED CT. Chang et al [36] reported the Pearson correlation to assess clinical semantic textual similarity, and the incorporation of SNOMED CT into ClinicalBERT improved the performance of the model by 1.77% and 2.36% using cui2vec [73] and KG embeddings, respectively.

**Table 5:** Percentage performance gain in classification tasks after SNOMED CT integration into large language models.

Studies			*F*_1_-score gain (%)		Precision gain (%)		Recall gain (%)		AUC^a^ gain (%)		Accuracy gain (%)
**Chopra et al [22]**			—^b^		—		—		—		+0.99
**Yadav et al [33]**			+26.05 (0.4718→0.5947)		+36.87 (0.4616→0.6318)		+16.41 (0.4826→0.5618)		—		+17.27 (0.4790→0.5617)
**Khosla et al [27]**			—		—		—		+0.85 (0.468→0.472)		—
**Zhang et al [48]**											
	BioBERT for intent detection	+1.15 (0.701→0.693)		—		—		—		—	
	Semantic matching for content recognition	—		−0.90 (1.000→0.991)		+12.15 (0.724→0.812)		—		—	
**Makhervaks et al [53]**											
	BERT based on MedNLI-General	—		—		—		+14.83 (0.661→0.759)		—	
	Bio-GPT on MedNLI-General	—		—		—		+10.34 (0.725→0.800)		—	

^a^AUC: area under the receiver operating characteristic curve.

^b^Not available.

##### MCN Tasks

The most prominent end task in NLU was MCN, with 15 studies involved. MCN, the task of linking textual mentions to concepts in an ontology, provides a solution for unifying different ways of referring to the same concept. All the studies approached concept recognition as a multilabel classification task involving entity extraction and entity typing from words, phrases, or sentences. Models were trained on corpora annotated with SNOMED CT concepts and semantic types to identify concept mentions and generate a list of candidate SNOMED CT concepts that best match those mentions from testing texts. When training from annotated corpora was not available, MetaMap [74] was used to extract biomedical entities mentioned in free texts and map them to ontology concepts [25,26,35,50]. When candidate concepts were ranked, representation vectors of mentions and concept descriptions were generated, and their similarity was calculated using cosine similarity [31,35,44,45,54], linear transformation such as support vector classifiers [52], or softmax function [23,41,43]. In a more rule-oriented approach, Borchert and Schapranow [47] calculated weights based on semantic type and preferred term status from a gazetteer to reorder candidate lists. In other studies [24,26,50], sieve-based multipass entity linking systems [75] were used to rank the most likely concepts and achieved superior performance compared to neural classifiers.

Most of the studies observed positive gains in accuracy in MCN tasks after SNOMED CT integration (Table 6). Two authors reported the pre- and postintegration *F*_1_-scores, recall values, and precision values and observed inconsistent results, with one reporting positive gains in the *F*_1_-score and precision value and the other demonstrating a loss in the *F*_1_-score and precision value after the integration of SNOMED CT.

**Table 6:** Percentage performance gain in medical concept normalization tasks after SNOMED CT integration into large language models.

Studies	*F*_1_-score gain (%)	Precision gain (%)	Recall gain (%)	Accuracy gain (%)
Peterson et al [25]	−1.05 (0.95→0.94)	−1.04 (0.96→0.95)	0 (0.94→0.94)	—^a^
Wang et al [26] (vs training data dictionary with exact match, ignore order “yes”)^b^	—	—	—	+27.36 (0.6013→0.7658)
Hristov et al [34]	—	—	—	+73.21 (0.56→0.97)
Dai et al (2021) [35]	—	—	—	+45.08 (0.417→0.605)
Xu and Miller [44] (on ShARe/CLEF 2013)	—	—	—	+0.68 (0.8333→0.8277)
Dong et al [54] (BLINKout on ShARe/CLEF 2013)	+5.87 (0.818→0.866)	+15.11 (0.741→0.853)	−3.62 (0.912→0.879)	+10.68 (0.777→0.860)

^a^Not available.

^b^The training data dictionary was constructed based on the Medical Concept Normalization corpus data. The SNOMED CT dictionary included the RxNorm dictionary.

#### NLG Tasks

##### Machine Translation

Several studies that participated in the WMT Biomedical Shared Task [76] described their methods for translating biomedical texts from various foreign languages, such as Spanish, French, German, and Chinese, as well as less-resourced languages, such as Basque, into English or vice versa. Transformer-based multilingual neural machine translation systems were the mainstream architectures, which were trained on dictionaries derived from SNOMED CT [28,30,39] or clinical notes artificially generated from SNOMED CT terminology contents [21,29].

The translation performance was reported using the Bilingual Evaluation Understudy (BLEU) score [77]. While most studies (4/5, 80%) presented improved BLEU scores by up to 131.66% [21] compared to their out-of-domain models, some studies (1/5, 20%) reported nonsuperior results [30] (Table 7).

**Table 7:** Performance comparison of biomedical translation tasks with and without SNOMED CT integration into large language models (LLMs).

Studies and translation direction			Performance on test data without SNOMED CT integration into an LLM (BLEU^a^ score)		Performance on test data with SNOMED CT integration into an LLM (BLEU score)		BLEU score gain after SNOMED CT integration into an LLM (%)
**Soto et al [21]**							
	Basque to Spanish	10.55		24.44		+131.66	
**Soto et al [30]**							
	Spanish to English	57.25		56.89		−0.63	
	English to Spanish	47.19		47.15		−0.08	
**Corral and Saralegi [29]**							
	English to Basque	12.85		13.61		+5.91	
**Peng et al [28]**							
	English to French	38.98		41.66		+6.88	
	French to English	38.31		38.44		+0.34	
**Wang et al [39]**							
	English to Italian	33.53		42.17		+25.77	
	Italian to English	36.43		43.72		+20.01	
	English to Portuguese	38.73		50.12		+29.41	
	Portuguese to English	41.84		54.74		+30.83	
	English to Russian	25.25		36.25		+43.56	
	Russian to English	39.76		47.09		+18.44	

^a^BLEU: Bilingual Evaluation Understudy.

##### Text Summarization

For medical text summarization, encoder-decoder LLMs were used to process input embeddings and produce simplified texts. Pattisapu et al [32] primarily focused on the simplification of verbose sentences. They substituted biomedical mentions with UMLS-preferred names and tokenized them at the subword level to produce noisy input sentences for training. In contrast, Searle et al [57] summarized entire hospital encounters into a few sentences by ranking the most salient ones to constitute the summary. To address the hallucination problem arising from LLMs, authors used SNOMED CT semantic tags of the extracted biomedical terms to configure guidance signals for clinical problems and interventions.

Recall-Oriented Understudy for Gisting Evaluation recall [78] measures how many n-grams in the source text appear in the summarization. Pattisapu et al [32] reported no gain in ROUGE recall when incorporating SNOMED CT into NLP pipelines. Searle et al [57] presented ROUGE-*F*_1_, a harmonized measure of the recall and precision for ROUGE, and observed improvements by 3.6% (from 11.1 to 11.5) and 48.84% (from 8.6 to 12.8) on the Medical Information Mart for Intensive Care III and King’s College Hospital corpora, respectively, after incorporating SNOMED CT.

##### Question Answering and Generation

Generating answers for short-answer or essay questions, as opposed to multiple-choice questions, can be classified as NLG. The task of question answering may involve preliminary NLU pipelines, such as intent and content recognition. Zhang et al [48] developed a clinical communication training dialogue system incorporated with SNOMED CT synonyms for the augmentation of textual data and BioBERT for intent recognition. They qualitatively evaluated the performance of the conversation system using scales rated by physicians from 29 training records, which indicated a comparable precision as clinical experts.

## Discussion

### LLMs and SNOMED CT

In this scoping review, we observed that BERT was the mainstream LLM integrated with SNOMED CT. Considering the significant time required to publish state-of-the-art methodologies, especially in peer-reviewed journals [79], it is unsurprising that more recent inventions, such as GPT-3.5 and BART, were less prevalent in articles published from 2018 to 2023. Researchers in this field exploited biomedically oriented BERT variants, such as BioBERT and PubMedBERT, reflecting the need for biomedical tasks to be trained or fine-tuned on specialized corpora [16]. However, due to privacy and confidentiality concerns, there is a dearth of clinical documents and patient notes, making it difficult to sufficiently train biomedical LLMs to an extent comparable to those in the general domain [80]. SNOMED CT can supplement or even substitute biomedical pretraining corpora, addressing the chronic shortage, as noted in this review. A substantial number of studies included in this review used SNOMED CT to expand pretraining corpora by concatenating synonyms or relations in documents or generating synthetic texts based on SNOMED CT descriptions or relations.

We identified 3 approaches to incorporating SNOMED CT into LLMs: LLM input, additional fusion modules, and knowledge retriever, with the former 2 intervening in the pretraining process of LLMs. While either lexical or graph information from SNOMED CT could be incorporated into the pretraining stage, the lexicon of SNOMED CT descriptions was the predominant form of integration. This underscores that SNOMED CT chiefly introduces synonym information to LLMs, yet relation information remains underused in NLP research. The advantage of SNOMED CT in defining relations between biomedical entities through semantic networks needs to be adopted for more sophisticated tasks such as knowledge inference and validation and highlighted within the biomedical NLP research community.

### End Tasks and Performance Reports

A significant number of studies included in this review engaged in the concept recognition process from free texts, whether as the final task or an intermediate step for subsequent tasks. Recognizing and extracting SNOMED CT concepts from the unstructured sections of EHRs is becoming crucial in clinical settings, where substantial patient information, such as social history and socioeconomic status, remains untapped in free-text clinical notes [81]. Leveraging previously unrepresented SNOMED CT concepts from free-text clinical data holds great potential in significantly enhancing clinical care and research, especially in the era of smart applications where patient-generated data can be integrated into EHRs through the representation of patient-authored texts with SNOMED CT concepts [82].

Only a small fraction of the included models disclosed performance comparisons before and after SNOMED CT integration. For example, only 6 (40%) out of 15 studies on MCN tasks provided information about the gain in the *F*_1_-scores or accuracy after SNOMED CT incorporation. This suggests that many biomedical NLP researchers do not focus on the role of SNOMED CT or other ontologies in improving their models. Moreover, some authors chose to demonstrate only selected metrics, potentially leading to publication bias that favors improved performance at first glance. In our review, we identified 7 studies that presented only 1 metric without disclosing others (excluding those that reported only the BLEU score, which is widely recognized as the best metric for measuring translation performance). This focus on a single metric may encourage researchers to optimize their models for that metric, potentially leading to underperformance in other areas. The NLP community needs to propose standardized methods for presenting performance and, if possible, develop new metrics that better reflect the specifics of NLU and NLG tasks performed by LLMs.

### Implications for Future Endeavors

The knowledge-intensive approaches to enhancing LMs, which are often renounced by those favoring deep learning–based approaches, still comprise a small portion of the artificial intelligence research community. However, in the face of immense computational power and the availability of data required by LLMs and deep learning–based systems, an increasing number of researchers now advocate the harmonization of the 2 approaches [83], and a plethora of KG-enhanced LLMs is developed in the general domain [10,84]. In addition to improving the performance of artificial intelligence models, ontologies and human-curated knowledge bases can address the explainability and controllability of artificial intelligence, probing facts within the human-interpretable form of system architectures [85]. Exploring the trade-offs in combining the 2 approaches is anticipated to contribute toward trustworthy and reliable artificial intelligence.

Among various biomedical terminology systems and ontologies, SNOMED CT was the primary focus in this review as a KG integrated with LLMs. Although the UMLS continues to dominate NLP research in the biomedical domain [16], SNOMED CT has the potential to expand its influence, given its governance over the health care industry. Consequently, the use of SNOMED CT as a reliable knowledge source becomes more feasible, considering its presence in various EHR systems or common data models. While this review did not identify real-world SNOMED CT–incorporated LLM applications directly tied to EHR systems, SNOMED CT is implicitly expected to support these systems as a standardized terminology system bound to syntactic interoperability structures such as FHIR and OpenEHR. In addition, medical institutions already implementing SNOMED CT in their EHR systems are anticipated to incorporate LLM applications and use SNOMED CT at the point of care [86]. Explicit descriptions of SNOMED CT in technical specifications or scientific papers by developers of these applications would have been valuable to include in this review.

### Limitations

One of the limitations of this scoping review is that we examined LLMs that accepted SNOMED CT only as a working ontology, leaving other biomedical ontologies out of our scope. To the best of our knowledge, however, there is no comprehensive review of the use of other biomedical ontologies within LLMs. The queries used in this review, especially the first one, retrieved articles that used a variety of biomedical ontologies, such as the UMLS, Medical Subject Headings, Gene Ontology, and Medical Wikidata. We chose to limit the scope of our review to SNOMED CT due to the heterogeneity of components among different ontology systems and the difficulty in delineating the contributions of each ontology in a standardized way. A more consolidated analysis of different ontologies used within LLMs awaits more comprehensive work.

A significant proportion of the included studies (23/37, 62%) were retrieved from conference proceedings. While we excluded short abstract articles and included only those that provided sufficient information to be categorized by our preset features, interested readers might find it challenging to delve into detailed methodologies from these proceedings articles. However, many of these papers refer to additional materials, such as GitHub (GitHub, Inc) repositories, to provide raw data and source codes; for example, Khosla et al [27] provided the source code of their system on GitHub [87]. We encourage more studies to share additional materials on open developer platforms to enhance methodology transparency and accelerate NLP research.

Another limitation of this review is that we could not conclude on how the integration of SNOMED CT improved the performance of LLMs. While most of the studies (14/18, 78%) observed a positive impact on performance after SNOMED CT integration, their statistical significance was not indicated. Moreover, the diversity of evaluation methods prevented us from performing a meta-analysis across all the included studies. While we examined whether SNOMED CT integration improved LLM performance by presenting percentage gains across various metrics, these results are prone to being misleading due to potential publication bias and the insufficient number of included studies. Nevertheless, this before-and-after comparison method, often adopted for comparative studies, effectively measures the effect of interventions (SNOMED CT in our case) within a single group or entity [88]. To control for confounding factors, we excluded models whose performance differences could be attributable to modalities other than SNOMED CT integration. For example, we excluded the study by Zotova et al [40] from our analysis because their performance might have been affected by the use of a different testing corpus. An evenhanded testing bed, such as a shared task competition under a single testing method requiring all participants to report performance differences before and after KG integration, could provide a controlled evaluation to reliably and objectively measure the contributions of KGs.

### Conclusions

In conclusion, this scoping review explored the methodologies and effectiveness of integrating SNOMED CT into LLMs. The predominant approach involved using SNOMED CT concept descriptions or graph embeddings as inputs for LM encoders, many of which were involved in MCN tasks. The endeavor to identify and extract SNOMED CT concepts from free texts was proven to be instrumental in enhancing the understanding and generation of NL texts for downstream tasks in the biomedical realm. However, our study revealed both a lack of standardized methods for assessing KG integration into LLMs and a scarcity of explicit performance reporting in existing research, highlighting significant gaps in current evaluation practices. These findings underline the need for more consistent reporting and evaluation practices in this field of research. Future research is anticipated to be more aware of the advantage of SNOMED CT when incorporating it into LLMs and to report findings in a manner that facilitates comparison across different works.

## Appendix

Multimedia Appendix 1 PRISMA-ScR (Preferred Reporting Items for Systematic Reviews and Meta-Analyses extension for Scoping Reviews) checklist.

Multimedia Appendix 2 Brief introduction to large language models.

Multimedia Appendix 3 Summary of the included studies.

## Abbreviations

BERT: Bidirectional Encoder Representations From Transformers
BLEU: Bilingual Evaluation Understudy
EHR: electronic health record
FHIR: Fast Healthcare Interoperability Resource
KG: knowledge graph
LLM: large language model
LM: language model
MCN: Medical Concept Normalization
NL: natural language
NLG: natural language generation
NLP: natural language processing
NLU: natural language understanding
PRISMA-ScR: Preferred Reporting Items for Systematic Reviews and Meta-Analyses extension for Scoping Reviews
UMLS: Unified Medical Language System

## Data Availability

The data analyzed during this study are available from the corresponding author on reasonable request.
