# Supplementary material for: Use of SNOMED CT in Large Language Models: Scoping Review
Source: JMIR Med Inform. 2024 Oct 7;12:e62924. doi: 10.2196/62924 (PMC11494256; doi:10.2196/62924)
Supplement: Multimedia Appendix 2 [file medinform_v12i1e62924_app2.pdf]

Multimedia Appendix 2

Brief Introduction to Large Language Models

Transformer-based large language models are advanced artificial intelligence systems that can understand and generate human-like text. These models have revolutionized natural language processing (NLP) and can perform a wide variety of tasks.

1. Natural Language Processing Tasks:

Transformer-based LLMs can handle numerous NLP tasks, including:

- Text generation (writing articles, stories, code)
- Translation between languages
- Summarization of long texts
- Question answering
- Sentiment analysis
- Named entity recognition
- Text classification
- Dialogue systems and chatbots
- Language understanding and inference

2. Key Components of Transformer Architecture:

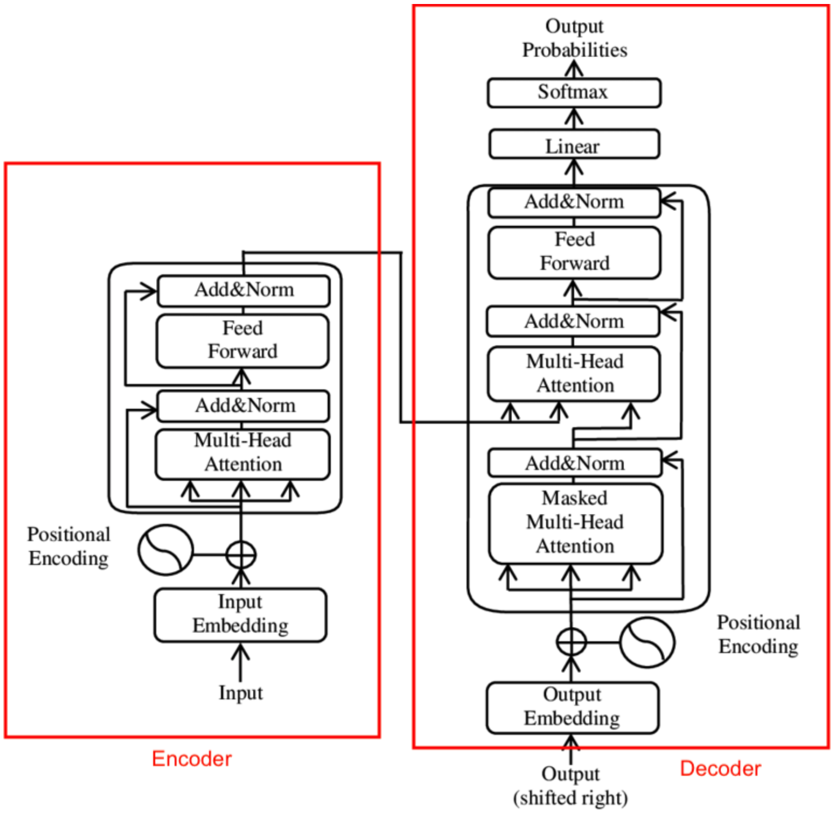

Figure S1. An illustration of main components of the transformer model. *Source*: Yuening Jia - DOI:10.1088/1742-6596/1314/1/012186

Encoders:

- Process input text by breaking it into tokens (words or subwords)
- Can look at the entire input sequence at once
- Build context-aware representations of each token
- Typically process tokens bidirectionally (left-to-right and right-to-left)

Decoders:

- Generate output text token by token
- Use previously generated tokens to predict the next one
- Usually process tokens unidirectionally (left-to-right)

Multi-Head Self-Attention Mechanism:

- Allows the model to focus on different parts of the input when processing each token
- Helps capture relationships between words, even if they're far apart in the text
- "Multi-head" means the model can pay attention to different aspects of the input simultaneously

Token Processing Direction:
- Encoders: Bidirectional (can look at entire input)
- Decoders: Unidirectional (looks at previous tokens to predict the next one)

3. Differences in Architecture: BERT, GPT, and BART

Model	Architecture	Input Processing	Output Generation	Primary Use Cases
BERT	Encoder-only	Bidirectional	N/A	Understanding tasks (e.g., classification, named entity recognition)
GPT	Decoder-only	Unidirectional	Left-to-right	Text generation tasks
BART	Encoder-Decoder	Bidirectional (encoder)	Left-to-right (decoder)	Both understanding and generation tasks

4. Brief Timeline of Natural Language Processing Models

Bag of words → Word embeddings → RNN-based models → LSTM-based models → Bi-directional LSTM → Attention-based models → Transformers

Figure S2. Timeline of natural language processing models.

"Bag-of-words" models
Bag-of-words (BOW) models are simple yet effective text representation techniques in natural language processing. They treat a document as an unordered collection of words, disregarding grammar and word order. Each unique word becomes a feature, and its frequency in the document is typically used as the feature value. However, they lose semantic information and context. Despite their limitations, BOW models serve as a foundation for more advanced text processing techniques and remain useful in many applications.

Word embeddings
Embedding models in natural language processing represent words, phrases, or documents as dense vectors in a continuous high-dimensional space. Unlike bag-of-words, embeddings capture semantic relationships between words, allowing similar concepts to be close in the vector space. Popular techniques include Word2Vec, GloVe, and FastText for word-level embeddings. Embeddings preserve semantic and syntactic information, enabling better performance in various NLP tasks such as similarity comparisons, clustering, and as input features for machine learning models.

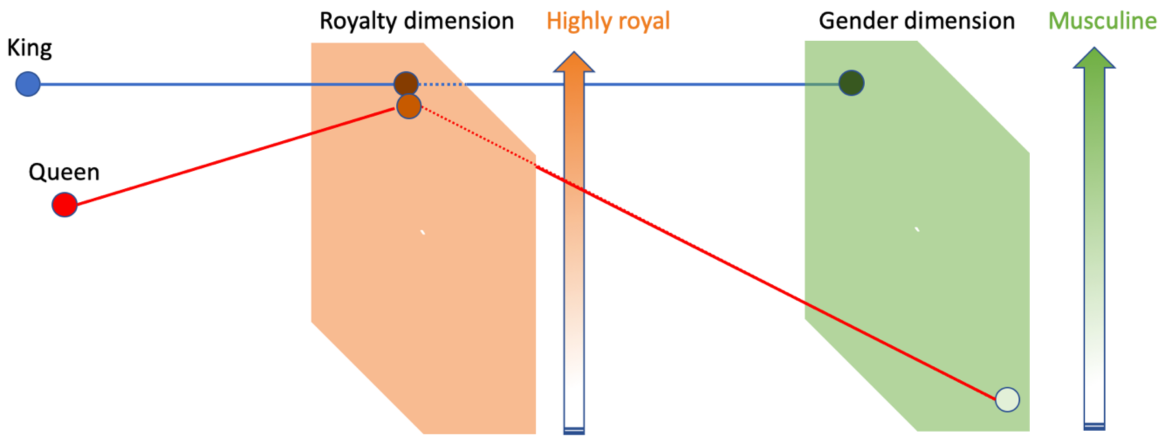

Figure S3. Illustration of how entities are projected to a vector space that each represent a dimension of meaning in word embedding models.

RNN based models

RNN-based NLP models use recurrent neural networks to process sequential text data. They maintain a hidden state that captures information from previous inputs, allowing them to handle variable-length sequences. RNNs can model context and dependencies in language, making them suitable for tasks like language modeling, machine translation, and sentiment analysis. However, they struggle with long-term dependencies due to vanishing gradients. Variants like LSTMs and GRUs were developed to address this limitation, improving performance on many NLP tasks.

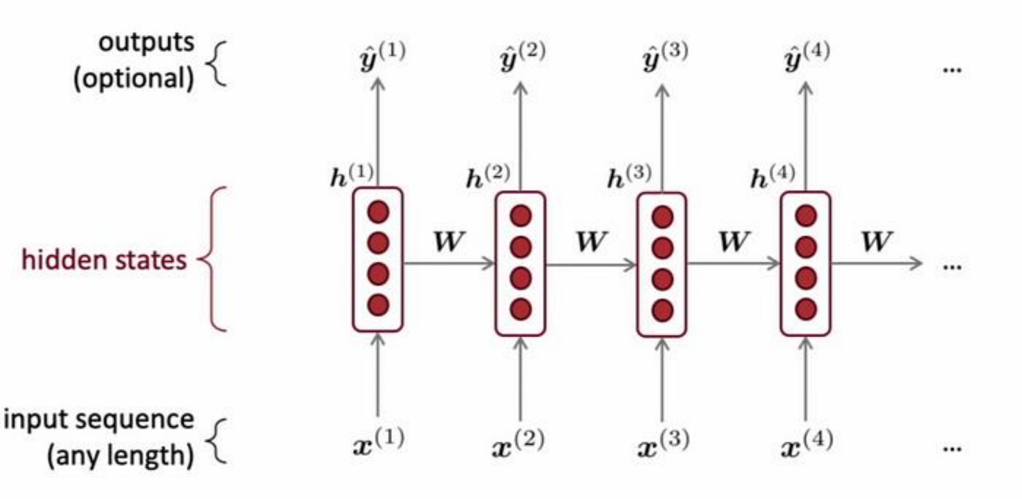

Figure S4. The architecture of vanilla RNN model. *Source*: Stanford CS224N course slides

LSTM-based models

LSTM-based models use Long Short-Term Memory networks, a type of RNN designed to handle long-term dependencies in sequential data. LSTMs use a cell state and gates (input, forget,

output) to control information flow. This architecture allows them to selectively remember or forget information, mitigating the vanishing gradient problem. LSTMs excel in tasks requiring long-range context understanding, such as language modeling, text generation, and sentiment analysis. They've been widely used in NLP applications before the advent of transformer models.

5. Advantages over Previous Models (RNNs and LSTMs):

Transformer-based LLMs differ from earlier models like Recurrent Neural Networks (RNNs) and Long Short-Term Memory networks (LSTMs) in several key ways:

Parallelization:
- RNNs and LSTMs process tokens sequentially, which is slower
- Transformers can process all input tokens in parallel, making them much faster to train and use

Long-range Dependencies:
- RNNs and LSTMs struggle with long-range dependencies in text
- Transformers can easily capture relationships between distant words thanks to the self-attention mechanism

Scalability:
- Transformers can be scaled to much larger sizes, allowing them to learn from vast amounts of data
- This scalability has led to the development of models with billions of parameters, capable of impressive language understanding and generation

Context Understanding:
- The self-attention mechanism allows transformers to build rich, context-aware representations of each token
- This results in better understanding of nuanced language use, idioms, and context-dependent meanings

No Vanishing Gradient Problem:
- RNNs and LSTMs can suffer from vanishing gradients, making it hard to learn from long sequences
- Transformers don't have this issue, allowing them to handle much longer texts effectively

Pre-training and Transfer Learning:
- Transformer models can be pre-trained on vast amounts of unlabeled text data
- This pre-training allows them to learn general language patterns and transfer this knowledge to specific tasks with minimal fine-tuning

Flexibility:

- The transformer architecture is highly adaptable to various NLP tasks
- Different configurations (encoder-only, decoder-only, or encoder-decoder) can be optimized for specific use cases

5. Summary

Transformer-based large language models represent a significant leap forward in NLP technology. Their ability to process text in parallel, capture long-range dependencies, and scale to enormous sizes has enabled them to achieve human-like performance on many language tasks. The flexibility of the architecture, as seen in models like BERT, GPT, and BART, allows for specialized models tailored to different types of NLP challenges. As these models continue to evolve, they are likely to play an increasingly important role in how we interact with and leverage textual information in the digital age.
